# CDs-Peroxyfluor Conjugation for Ratiometric Fluorescence Detection of Glucose and Shortening Its Detection Time from Reaction Dynamic Perspective

**DOI:** 10.3390/bios13020222

**Published:** 2023-02-03

**Authors:** Yangjie Li, Site Luo, Xin Wang, Yang He, Haihu Yu

**Affiliations:** National Engineering Laboratory for Fiber Optic Sensing Technology, Wuhan University of Technology, Wuhan 430070, China

**Keywords:** carbon dots, ratiometric fluorescence, glucose, rapid detection, fluorescein, kinetics

## Abstract

A ratiometric fluorescence probe based on the conjugation of peroxyfluor-NHS (PF) and carbon dots (CDs) was designed for selective and rapid detection of glucose. When glucose was catalytically oxidized by glucose oxidase (GOx), the product H_2_O_2_ would react with colorless and non-fluorescent peroxyfluor moiety to give the colored and fluorescent fluorescein moiety which would absorb the energy of CDs emission at 450 nm due to the Förster Resonance Energy Transfer (FRET) and generate a new emission peak at 517 nm. The reaction between PF and H_2_O_2_ was slow with a rate constant of about 2.7 × 10^−4^ s^−1^ under pseudo-first-order conditions (1 uM PF, 1 mM H_2_O_2_), which was unconducive to rapid detection. Given this, a short time detection method was proposed by studying the kinetics of the reaction between PF and H_2_O_2_. In this method, the detection time was fixed at three minutes. The linear detection of glucose could be well realized even if the reaction was partially done. As glucose concentration increased from 0.05 mM to 5 mM, the fluorescence intensity ratio (I_517_/I_450_) after 3 minutes’ reaction of CDs-PF and glucose oxidation products changed linearly from 0.269 to 1.127 with the limit of detection (LOD) of 17.19 μM. In addition, the applicability of the probe in blood glucose detection was verified.

## 1. Introduction

Diabetes is a serious chronic condition afflicting hundreds of millions of people. It is among the top 10 causes of death in adults, and 4.2 million people among 20–79-year-old adults were estimated to die from diabetes and its complications in 2019 [1,2]. Detecting and controlling the blood glucose of diabetic patients will limit the long-term damage of diabetes to the heart, eyes, kidneys, nerves and other organs and reduce risk of premature death [3,4]. Therefore, the development of an efficient and accurate method for blood glucose monitoring is important for the treatment of diabetic patients and the detection of prediabetic individuals. A variety of methods for glucose detection have been reported in the past few years, such as fiber-optic surface plasmon resonance [5,6,7,8], electrochemical [9,10,11,12,13], surface-enhanced Raman scattering [14], electrochemiluminescence [15], fluorescence [16,17,18,19] and so on. Among them, the fluorescence method has received a lot of attention because of its simplicity, efficiency and sensitivity. Moreover, if two appropriate fluorophores are combined a ratiometric fluorescence probe possessing self-calibration will be established to eliminate the instrument errors and external interference to some extent, thus improving the detection accuracy.

As novel fluorescent materials, carbon dots (CDs) have great application prospects in biosensing due to their high quantum yield, biocompatibility, low toxicity, low cost and stable chemical property [20,21,22,23,24]. CDs have been used for detections of analytes such as metal ions [25] and small organic molecules [26,27,28]. However, most CDs-based probes have single-emission detection, which brings many instrument errors to the detection and requires frequent calibrating [25,28]. CDs-based ratiometric fluorescence probes with dual-emission solved the problem as the errors could be eliminated by self-calibrating [29,30]. CDs-based ratiometric fluorescence probes for glucose detection have been widely studied. Cui et al. demonstrated noninvasive ratiometric fluorescence detection of sweat glucose using a wearable skin pad based on luminescent porous silicon decorated with CQDs [30]. However, the slow reaction between luminescent porous silicon and H_2_O_2_ made the method time-consuming. Liu et al. proposed ratiometric fluorescence and colormetric sensing system for the detection of H_2_O_2_ and glucose [16], but introducing several intermediaries rather than directly measuring H_2_O_2_ brought many error factors to the sensing system. Many fluorescence probes for glucose detection are based on the detection of H_2_O_2_ produced by oxidation of glucose. However, the slow reaction between probe and H_2_O_2_ limits their development and the selectivity of the probes for H_2_O_2_ needs to be improved. 

**Scheme 1:**
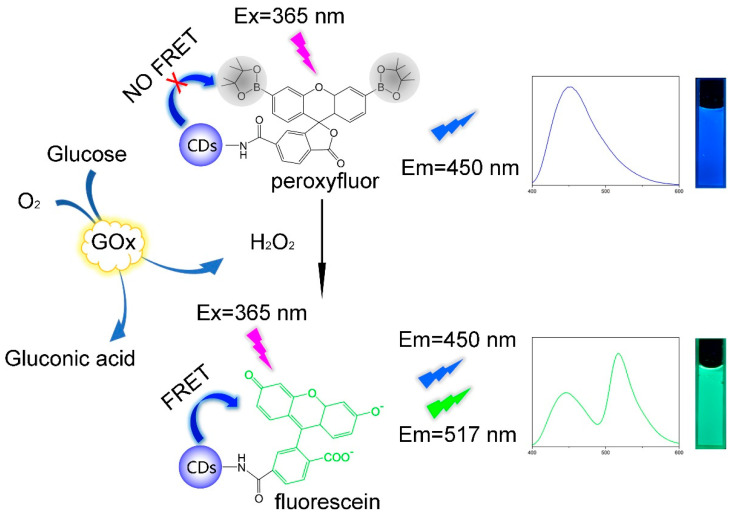
Schematic illustration of ratiometric fluorescence detection of glucose based on CDs-PF.

Here we proposed a novel ratiometric fluorescence probe for selective and rapid detection of glucose which combined CDs and PF. The probe is highly selective for H_2_O_2_ because of the selective H_2_O_2_-mediated transformation of arylboronates to phenols [31]. The selectivity of PF for H_2_O_2_ over other ROS relies on deprotection rather than oxidation, which makes PF an excellent H_2_O_2_ probe [32]. On the other hand, we have shortened the detection time to 3 min from reaction dynamic perspective. CDs and fluorescein are an ideal FRET pair for ratiometric fluorescence probes because of the large spectral overlap of the fluorescein absorption and the CDs emission [29]. The fluorescein is widely used as chemosensors because of its high extinction coefficients, excellent quantum yields, great photostability and relatively long emission wavelengths [33]. To our best knowledge, there are few studies on glucose detection based on CDs-Fluorescein. As shown in Scheme 1, when glucose was catalytically oxidized by glucose oxidase (GOx), the product H_2_O_2_ would react with colorless and non-fluorescent peroxyfluor moiety to give the colored and fluorescent fluorescein served as the acceptor corresponding to the donor of CDs in the FRET pair. The newly produced fluorescein would quench the blue fluorescence of CDs at 450 nm and generate a new green emission at 517 nm simultaneously, allowing the ratiometric fluorescence detection of glucose. However, the reaction between PF and H_2_O_2_ is too slow, which limits its application in biosensors. Aaron E. Albers et al. had first proposed the PF-based ratiometric fluorescence probe for H_2_O_2_ detection [34], but they did not realize quantitative and rapid detection of H_2_O_2_. Consequently, we changed our perspective by focusing on the reaction process rather than the reaction endpoint. By studying the kinetics of the reaction between PF and H_2_O_2_, the total reaction order is confirmed to be 2, and the reaction order of both components are 1. On this basis, we proposed an improved method: the reaction time of the probe and glucose oxidation products was fixed at 3 min; that is to say, the fluorescence intensity of the mixture after 3 min is taken as the detection result in a certain glucose concentration. Experimental results demonstrated that fluorescence intensity ratio (I_517_/I_450_) after 3 minutes’ reaction changed linearly with the glucose concentration, even if the reaction was partially done. The rapid detection of glucose based on CDs-PF was well realized by the method of recognizing the whole through observation of the part. Shortening detection time is meaningful as we can acquire the glucose level in several minutes rather than several hours. The method has potential in extending to other time-consuming detections in biosensing.

## 2. Experimental Section

### 2.1. Reagents and Instruments

D-glucose, hydrogen peroxide (30%) and citric acid were purchased from Sinopharm Chemical Reagent Co. Glucose oxidase (GOx), 3-Bromophenol, anhydrous N, *N*′-dimethylformamide (DMF), *N*-(2-Aminoethyl)-3-aminopropyltrimethoxysilane (AEAPTMS), 1,2,4-benzenetricarboxylic acid, dichloro [1,1′-bis(diphenylphosphino) ferrocene] palladium (II), methanesulfonic acid, potassium acetate and bis(pinacolato) diboron were purchased from Aladdin Reagent Co., Ltd. (Shanghai, China). Phosphate Buffered Saline (0.1 M, pH = 7.4) was prepared by disodium hydrogen phosphate (Na_2_HPO_4_) and sodium dihydrogen phosphate (NaH_2_PO_4_). All the reagents were analytical grade and used without further purification, and the water used in the experiments w ultrapure water produced with a Hitech-K flow water purification system.

The emission spectra of the probe were recorded by a F-4500 FL Spectrophotometer (Hitachi, Japan). The absorption spectra were measured by a UV-2450 UV–visible spectrophotometer (Shimadzu Co., Ltd., Tokyo, Japan). The transmission electron microscopy (TEM) images were observed by the JEM-2100F (JEOL Ltd., Japan). The element composition of the compound was characterized by X-ray photoelectron spectroscopy (ESCALAB 250Xi, Thermo Fisher Scientific, Waltham, MA, USA). Fourier transform infrared (FT-IR) spectrum was collected with a Nexus spectrometer. The fluorescence lifetime was collected with a FluoroMax-4 fluorescence spectrometer (HORIBA Scientific, Piscataway, NJ, USA). All fluorescence photographs were obtained with a Nikon D5600 digital camera.

### 2.2. Synthesis of the CDs-PF Composite Probe

CDs and 3′,6′-dibromofluoran-6-carboxy succinimidyl ester were synthesized according to the previously reported method [34,35]. Then, 30.9 mg of 3′,6′-dibromofluoran-6-carboxy succinimidyl ester, 72 mg of bis(pinacolato) diboron, 30 mg of potassium acetate and 5 mL of dry DMF were added in a Schlenk tube. The reaction was heated at 80 °C in an oil bath pan for 5 h under a nitrogen atmosphere. The resulting dark brown reaction was cooled to room temperature and poured into 50 mL of acidic ice water to precipitate a dark brown solid. The solid was then dissolved in dichloromethane and eluted through a silica-gel column with 1:3:100 of methanol/acetic acid/dichloromethane to afford Peroxyfluor-NHS (PF) as a greenish solid.

The composite probe CDs-PF was prepared as follows: Peroxyfluor-NHS would react with the amino on the surface of CDs to prepare the CDs-PF composite probe; 10 mg of Peroxyfluor-NHS was dissolved in 1 mL of dry DMF in a flask; 100 μL of CDs fluid in 1 mL of dry DMF was added dropwise to the PF/DMF solution; and the reaction was stirred at room temperature for 12 h in the dark. A large quantity of brown precipitate was observed in the flask, which was collected by suction filtration and washed with DMF three times. The resultant solid was eluted through a silica-gel column with 1% methanol and dichloromethane to afford the CDs-PF as brown solid.

### 2.3. Quantum Yield (QY) Measurements

Fluorescence quantum yield was estimated using the quinine sulfate reference method. Quantum yield of standard solution of quinine sulfate in 0.1 M H_2_SO_4_ was 54%. The fluorescence quantum yield can be calculated using the following equation:

(1) $$Y_{u}=Y_{s}\times \frac{F_{u}}{F_{s}}\times \frac{A_{s}}{A_{u}}$$

where *Y_u_* and *Y_s_* are the fluorescence quantum yield of CDs and quinine sulfate, respectively, *F_u_* and *F_s_* are the integrated fluorescence intensity of CDs and quinine sulfate, respectively, and *A_u_* and *A_s_* are the absorbance of CDs and quinine sulfate under the same excitation wavelength.

### 2.4. Kinetics Experiments of PF with H_2_O_2_

There are two parts of the kinetics experiments. In the first part, the probe solution with concentration of 0.1 mM was prepared by dissolving PF in PBS (0.1 M, pH = 7.4). The H_2_O_2_ solution was prepared with the concentration range from 0.1 M to 3 M. The fluorescence detection would be carried out every 10 s for about 5 min as soon as 3 mL of the probe solution and 3 μL of the H_2_O_2_ solution were mixed in the cuvette. In the second part, PF and H_2_O_2_ were exchanged. The concentration of H_2_O_2_ was fixed at 1 mM in 3 mL of probe solution, and the concentration of PF changed from 0.01 mM to 0.2 mM. The later experiment is the same as part 1.

### 2.5. Ratiometric Fluorescence Detection of Glucose

The composite probe with concentration of 0.075 mg/mL was prepared by dissolving CDs-PF in PBS (0.1 M, pH = 7.4). The ratiometric fluorescence detection of glucose was based on enzyme catalysis. Various concentrations of glucose solution were incubated with 0.2 mg/mL of glucose oxidase in PBS (0.1 M, pH = 7.4) at 37 ℃ for 40 min. Then, 1 mL of the solution to be detected was mixed with 2 mL of the probe solution. The fluorescence intensity was recorded after reaction for 3 min for every detection.

### 2.6. Real Sample Detection

Firstly, the blood samples were centrifuged at 10,000 rpm for 20 min. The collected serum samples were diluted 100-fold with PBS buffer (0.1 M, pH = 7.4). Then the diluted serum samples were spiked with different concentrations of glucose. Finally, the glucose of the spiked serum samples was detected by the ratiometric fluorescence detection method previously mentioned.

## 3. Results and Discussion

### 3.1. Characterization of the CDs-PF

**Figure 1:**
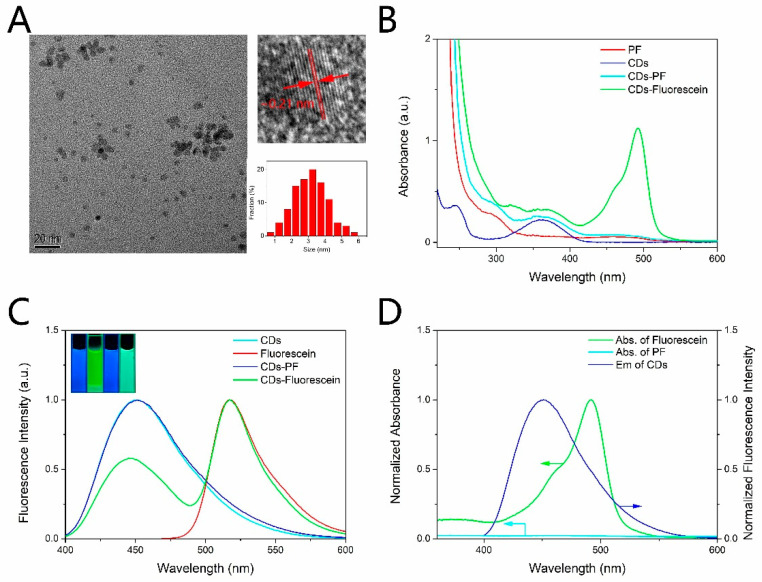
(**A**) TEM image, HRTEM image and size distribution of CDs-PF. (**B**) UV–vis absorption spectra of PF, CDs, CDs-PF, CDs-Fluorescein. (**C**) Fluorescence spectra of CDs, CDs-PF, fluorescein, and the dual emission of CDs-Fluorescein. Excitation was at 365 nm for CDs, CDs-PF, CDs-Fluorescein, and 460 nm for Fluorescein. Inset: fluorescence images of CDs, fluorescein, CDs-PF, CDs-Fluorescein under illumination of UV light (365 nm). (**D**) Spectral overlap of fluorescein absorption and the CDs emission, and the small overlap of PF absorption and the CDs emission.

The morphology of CDs was observed by a transmission electron microscopy (TEM). The TEM image (Figure 1A) showed that CDs-PF were monodispersed with the diameter distributed in the range from 1 nm to 5 nm and the average diameter of ~3.1 nm. Moreover, the clear lattice fringes with a spacing of 0.21 nm in accordance with the (100) facets of graphite were observed in the HRTEM image [36]. Fourier transform infrared (FT-IR) spectra were measured to confirm the combination of PF and CDs. The FT-IR spectra of CDs, PF, CDs-PF and CDs-Fluorescein were shown in Figure S1 (in Supplementary Material). The broad peak at 3347 cm^−1^ of CDs is attributed to O-H and N-H stretching vibrations demonstrating the presence of hydroxyl and amino groups [37]. The existence of the amino makes the amide reaction between CDs and peroxyfluor-NHS possible, while the deeper peak of CDs-PF at 1671 cm^−1^ is assigned to the amide bonding, indicating the conjugation of CDs and PF. There are no apparent FT-IR changes between CDs-PF and CDs-Fluorescein, which may be attributed to the low content of the PF moiety. The element composition of the compound was characterized by X-ray photoelectron spectroscopy. The XPS spectrum (Figure S2) showed that CDs-PF was composed of carbon (71.79%), nitrogen (8.54%), oxygen (16.77%), boron (2.9%). A small amount of boron further confirms the conjugation of CDs and PF (Figure S2).

### 3.2. Optical Properties of CDs, PF, CDs-PF, CDs-Fluorescein

Fluorescence spectra and absorption spectra of CDs, PF, CDs-PF, CDs-Fluorescein were measured to study their optical properties. As shown in Figure 1B, there are two distinct absorption peaks at 245 nm and 360 nm in the UV–vis absorption spectrum of CDs. The peak at 245 nm is attributed to the $\pi \to \pi^{*}$ transition of the aromatic C=C bonds, which leads to no fluorescence. The other peak at 360 nm is attributed to the complicated surface states, which results in strong emission [38]. CDs exhibited strong blue fluorescence centered at 450 nm under the illumination of UV light (365 nm), as shown in Figure 1C. CDs have the same emission wavelength when they are excited under excitation wavelength ranging from 320 nm to 420 nm (Figure S3). The excitation independence can be attributed to the surface molecular state emission of CDs [36]. What is more, CDs have good photostability as there are no apparent fluorescence intensity changes when CDs were exposed to UV light (365 nm) for 90 min (Figure S4). PF has no obvious absorption peak in visible band (Figure 1B), and it showed no fluorescence under UV light and visible light as the xanthenone scaffold is protected by the boronate groups to adopt a non-fluorescent lactone form [22]. After reacting with H_2_O_2_, PF transformed to the fluorescein which exhibited bright green fluorescence centered at 517 nm under UV light excitation (Figure 1C), and a distinct absorption peak at 490 nm appeared (Figure 1B). Figure 1D shows a large spectral overlap of fluorescein absorption and the CDs emission, which makes FRET possible. As shown in Scheme 1, in the absence of H_2_O_2_ FRET was inhibited because of the small overlap of the acceptor moiety absorption and the donor moiety emission (Figure 1D), and CDs-PF exhibited only blue fluorescence centered at 450 nm under the illumination of UV light (365 nm) (Figure 1C). Upon treatment with H_2_O_2_, a dual-emission centered at 450 nm and 517 nm was observed (Figure 1C), corresponding to the emission of CDs moiety and fluorescein moiety, respectively. Newly produced fluorescein moiety showed strong absorption in the CDs emission region. Due to the activation of FRET, as the reaction proceeds, the emission peak at 517 nm increased and the emission peak at 450 nm decreased, as shown in Figure S5. At the same time, the color of the fluorescence of the solution under UV light changed from blue to green. Furthermore, the fluorescence properties of CDs and fluorescein are sensitive to pH, as shown in Figure S6. The fluorescence of both CDs and fluorescence was weakened in the acidic conditions, and the emission of CDs showed red shift with pH decreasing. The protonation and deprotonation of CDs results in the pH-dependence [39], and the pH-dependence of fluorescein is attributed to the presence of three acid-base equilibria in different pH [40]. The QY of the synthesized CDs was estimated to be 81.5%.

### 3.3. Detection Mechanism of the Probe

The mechanisms of glucose detection are based on the response of the CDs-PF to H_2_O_2_ which is attributed to the H_2_O_2_-mediated transformation of arylboronates to phenols [31], as Scheme 1 indicated. In the absence of H_2_O_2_, boronic ester groups at the 3′ and 6′ positions of a xanthenone scaffold would force the PF moiety to adopt a colorless and non-fluorescent lactone form. Only blue fluorescence was observed as Figure S7 showed. As glucose was catalytically oxidized to give H_2_O_2_, H_2_O_2_-mediated electrophilic substitution in the benzene ring occurred and the boronic ester groups were replaced by hydroxy. Thus, the colored and fluorescent fluorescein generated with hydrolytic deprotection of the boronates. Then, FRET was activated. As a result, the fluorescence intensity at 517 nm increased and the fluorescence intensity at 450 nm decreased as shown in Figure S7. As a result, it was possible to detect glucose concentration through recording the fluorescence intensity of the probe. The binding affinity of PF and H_2_O_2_ was calculated by measuring the dissociation constants (*Kd*) of PF in the presence of H_2_O_2_ as previous work did [41,42]. Fluorescence intensities were recorded as the PF concentration changed from 0 to 300 μM in the presence of H_2_O_2_ (Figure S8). The Kd value was calculated to be 18.7 μM.

To further confirm the occurrence of the FRET mechanism in the CDs-PF platform, the Förster distance (*R*_0_), energy transfer efficiency (*E*), and the distance between the acceptor and the donor (*r*) were calculated by equation as follows [43]:

(2) $$E=\frac{R_{0}^{6}}{R_{0}^{6}+r^{6}}$$

(3) $$R_{0}=0.211{\left(\kappa^{2}n^{-4}\Phi J(\lambda )\right)}^{\frac{1}{6}}$$

where $\kappa^{2}$ is the orientation factor of the donor and acceptor transition dipoles and is typically assumed to be 2/3, Φ is the quantum yield of the donor (=81.5%), *n* is the refractive index of the medium (=1.33). *J(λ)* is the integral of overlap values which is expressed as:

(4) $$J(\lambda )=\int_{n}^{\infty }F_{D}(\lambda )\varepsilon_{A}(\lambda )\lambda^{4}d_{\lambda }$$

where $F_{D}(\lambda )$ is the donor normalized fluorescence emission spectrum, $\varepsilon_{A}(\lambda )$ is the acceptor molar absorption coefficient, and λ is the wavelength. After calculating, we obtained that *R*_0_, *E* and *r* were 1.71 nm, 20%, 2.15 nm, respectively. *R*_0_ and *r* were in the range of 1–10 nm, which was consistent with requirements for FRET mechanism [44].

Fluorescence lifetime was also measured to demonstrate the FRET occurred in the CDs-PF platform. Figure S9 and Table S1 show the fluorescence lifetime at 450 nm of CDs-PF and CDs-Fluorescein. The lifetime of the CDs decreased from 13.16 ns to 9.27 ns upon the transformation of the acceptor moiety from PF to fluorescein, implying the existence of FRET between CDs moiety and fluorescein moiety [45].

### 3.4. Kinetics of the Reaction between PF and H_2_O_2_

The kinetics of the reaction between PF and H_2_O_2_ are applicable to the CDs-PF probe as CDs moiety does not affect the reaction. The reaction equation is simplified as follows, as the intermediate reaction is rapid [31], F is the produced fluorescein, and the unimportant products are omitted:

(5) $$\mathrm{PF}+2H_{2}O_{2}\to F$$

The reaction rate can be expressed as:

(6) $$v=-\frac{dc_{\mathrm{PF}}}{dt}=\frac{dc_{F}}{dt}=kc_{\mathrm{PF}}{}^{n_{1}}c_{H_{2}O_{2}}{}^{n_{2}}$$

where $c_{\mathrm{PF}}$ and $c_{H_{2}O_{2}}$ are the concentrations of PF and H_2_O_2_ in the reaction process, respectively, and $c_{\mathrm{PF},0}$, $c_{H_{2}O_{2},0}$, $c_{F}$ in the later formula are the initial concentrations of PF, H_2_O_2_ and the concentration of the produced fluorescein. The *k* is the reaction rate constant and *n*_1_, *n*_2_ are the reaction order of each component. Under initial reaction conditions, the natural logarithm of both sides of the Formula (6) is taken to obtain the following formula:

(7) $$\ln v_{0}=\ln k+n_{1}\ln c_{\mathrm{PF},0}+n_{2}\ln c_{H_{2}O_{2},0}$$

The $v_{0}$ is the initial reaction rate equal to the formation rate of fluorescein and the consumption rate of PF under initial reaction conditions. In fact, the fluorescence intensity of fluorescein is linear dependent on its concentration in the low concentration range (dozens of μM), as indicated in Figure S10. This study is carried out under initial reaction conditions and is applicable to low concentration conditions. Therefore, the formation rate of fluorescein can be represented by the change rate of the fluorescence intensity.

**Figure 2:**
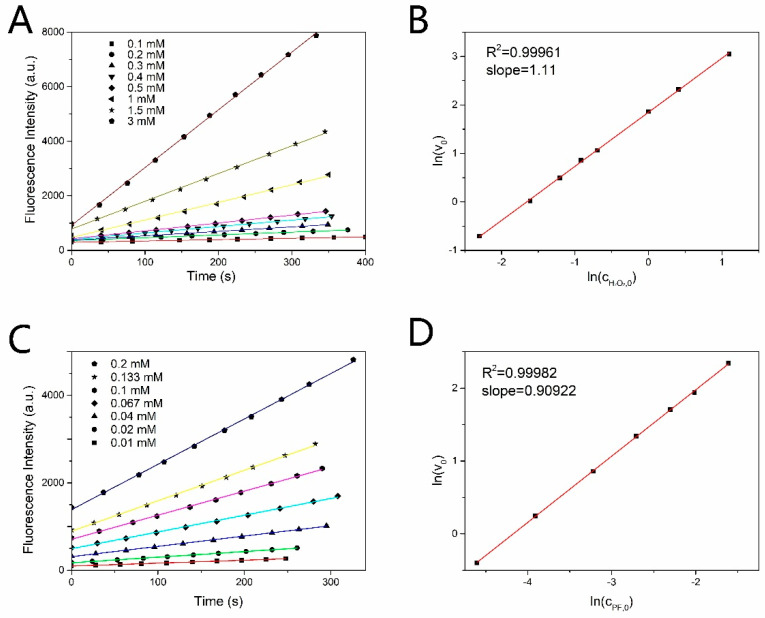
(**A**) Time response of fluorescence intensity of produced fluorescein in the actual H_2_O_2_ concentration range of 0.1 mM−3 mM with fixed PF concentration. (**B**) Linear relation between $\ln c_{H_{2}O_{2},0}$ and $\ln v_{0}$. (**C**) Time response of fluorescence intensity of produced fluorescein in the PF concentration range of 0.01 mM−0.2 mM with fixed concentration of H_2_O_2_. (**D**) Linear relation between $\ln c_{PF,0}$ and $\ln v_{0}$.

**Figure 3:**
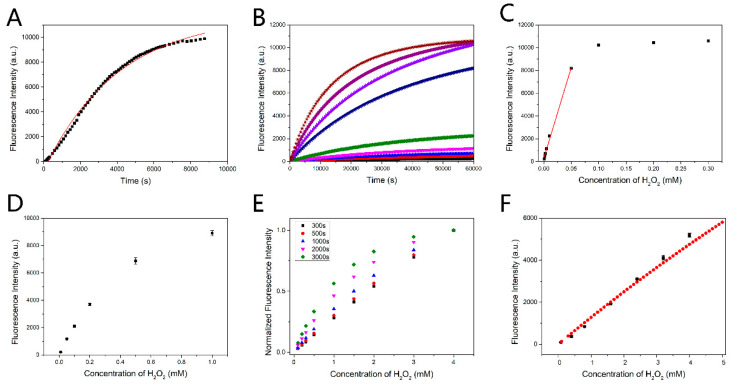
(**A**) Time-course kinetic measurement of the fluorescence response of PF to H_2_O_2_. Data were collected under pseudo first-order conditions (1 μM PF, 1 mM H_2_O_2_). Excitation was at 460 nm. The fluorescence peak was recorded every 2 min. (**B**) Time response of the probe in different H_2_O_2_ concentrations (0.001, 0.002, 0.003, 0.005, 0.01, 0.05, 0.1, 0.2 and 0.3 mM). The probe concentration was 50 μM. (**C**) 60,000 s fluorescence intensity followed the change in the H_2_O_2_ concentration corresponding to the (**B**). (**D**) 20,000 s fluorescence intensity followed the change in the H_2_O_2_ concentration (0.01, 0.05, 0.1, 0.2, 0.5 and 1 mM). The concentration of PF was 0.5 mM. (**E**) The theoretical detection results of PF to H_2_O_2_ were acquired from the rapid detection method when detection time was fixed at 300 s, 500 s, 1000 s, 2000 s and 3000 s. The concentration of PF was set as 70μM. In addition, 0.27 s^−1^·M^−1^ was taken as the rate constant *k*. The linearity decreased with the detection time increasing. (**F**) The theoretical linear detection result (red) and the experimental result (black). The concentration of PF was 0.1 mM in the theoretical detection and the actual detection 0.27 s^−1^·M^−1^ was taken as the rate constant *k* in the theoretical detection. The relationship between concentration and fluorescence intensity in the theoretical detection referred to the result of Figure S10. Error bars in (**D**) and (**F**) are based on standard deviations (n = 3).

The reaction order n_1_ and n_2_ were measured by controlling variable method based on the formula (7). When the initial concentration of PF was fixed at 0.1 mM, $v_{0}$ (determined by the slope) changed with the concentration of H_2_O_2_ as shown in Figure 2A. The $\ln v_{0}$ changed linearly with $\ln c_{H_{2}O_{2},0}$ as shown in Figure 2B. The slope of the line in Figure 2B is the reaction order of H_2_O_2_. Similarly, fixing the initial concentration of H_2_O_2_ at 1 mM, $v_{0}$ and the linear relationship between $\ln v_{0}$ and $\ln c_{\mathrm{PF},0}$ were shown in Figure 2C,D, respectively. The reaction order of H_2_O_2_ and PF are both 1 by taking the nearest integer. The reaction rate constant was estimated to be 0.27 s^−1^·M^−1^ by exponential decay fitting curve, as shown in Figure 3A.

### 3.5. Rapid Detection Principle

Rapid detection is significant for biosensing. In general, the results of the detection are taken from reaction endpoint. However, the reaction between PF and H_2_O_2_ is slow, requiring 2 h for 90% reaction accomplishment, as shown in Figure 3A (1 μM PF, 1 mM H_2_O_2_). We recorded the time response of the probe in different H_2_O_2_ concentrations. The PF concentration was 50 μM. The fluorescence intensity of the reaction endpoint (60,000 s) changing with H_2_O_2_ is shown in Figure 3B. The linear response of the fluorescence intensity to H_2_O_2_ only occurred in low concentration range, as the probe would react completely with H_2_O_2,_ and the fluorescence intensity changed little when the H_2_O_2_ concentration was greater than half of the PF concentration (Figure 3C). On the other hand, increasing the PF concentration would bring another problem: the fluorescence intensity and the fluorescein concentration will deviate from the linear relation when the reaction product fluorescein concentration is high (Figure 3D). Thus, it is time-consuming if the reaction endpoint is taken as the result and only low concentration H_2_O_2_ can be detected. Therefore, we proposed an improved method for rapid detection. It should be noted that this method is applicable for low concentration of the fluorescein conditions.

The total reaction order was confirmed to be 2, and the order of both components was 1. Therefore, the reaction rate can be expressed as:

(8) $$-\frac{dc_{\mathrm{PF}}}{dt}=kc_{\mathrm{PF}}c_{H_{2}O_{2}}$$

and we can derive another formula based on stoichiometry of the reaction Equation (5):

(9) $$\frac{c_{\mathrm{PF},0}-c_{\mathrm{PF}}}{c_{H_{2}O_{2},0}-c_{H_{2}O_{2}}}=\frac{1}{2}$$

Combining the Formulas (8) and (9), we get a new expression of the rate:

(10) $$-\frac{dc_{\mathrm{PF}}}{dt}=kc_{\mathrm{PF}}(2c_{\mathrm{PF}}+(c_{H_{2}O_{2},0}-2c_{\mathrm{PF},0}))$$

Then, integrating the above formula to give the following formula:

(11) $$\frac{1}{c_{H_{2}O_{2},0}-2c_{\mathrm{PF},0}}\ln(\frac{c_{H_{2}O_{2}}/c_{H_{2}O_{2},0}}{c_{\mathrm{PF}}/c_{\mathrm{PF},0}})=kt$$

At last, combining the Formulas (9) and (11) with the produced fluorescein concentration expression $c_{F}=c_{PF,0}-c_{PF}$, we get a function to express the produced fluorescein concentration as follows:

(12) $$c_{F}=\frac{c_{\mathrm{PF},0}c_{H_{2}O_{2},0}-c_{H_{2}O_{2},0}c_{\mathrm{PF},0}\exp(kt(c_{H_{2}O_{2},0}-2c_{\mathrm{PF},0}))}{2c_{\mathrm{PF},0}-c_{H_{2}O_{2},0}\exp(kt(c_{H_{2}O_{2},0}-2c_{\mathrm{PF},0}))}$$

It can be seen that the fluorescein concentration $c_{F}$ is determined by three parameters, $c_{PF,0}$, $c_{H_{2}O_{2},0}$ and *kt*, among which $c_{PF,0}$ is the initial concentration of the probe PF with a fixed value in the detection. On the other hand, $c_{F}$ can be expressed as $c_{F}=mFi$, where *m* is the proportionality coefficient and *Fi* is the fluorescence intensity of the fluorescein in the low concentration conditions. If *kt* is fixed, the Formula (12) will become a monotonic function of $c_{H_{2}O_{2},0}$. Thus, the concentration of H_2_O_2_ to be detected is expressed by the fluorescence intensity of the produced fluorescein which can be directly measured. Fixing the detection time *t* at a short time scale, the fitting curve of the right side of the Formula (12) is quasi-linear. Therefore, the fluorescence intensity changes linearly with the initial concentration of H_2_O_2_ because of the linear correlation between $c_{F}$ and the fluorescence intensity. The optimal detection time was determined as 3 min, as the fitting curve becomes nonlinear in longer detection time (Figure 3E) and more errors occur in shorter detection time. About 1.5% of the reaction was accomplished after 3 min reaction. The fluorescein derivative concentration exactly accords with the low concentration condition. The method shortens detection time in comparison with the general detection method in which several hours is needed. The experimental results agree well with the theoretical results as shown in Figure 3F, which indicates the feasibility of the method. With the catalytic oxidation of glucose by GOx, rapid and linear detection of glucose can be well realized by this method.

### 3.6. Ratiometric Fluorescence Detection of Glucose and Selectivity of the Probe

**Figure 4:**
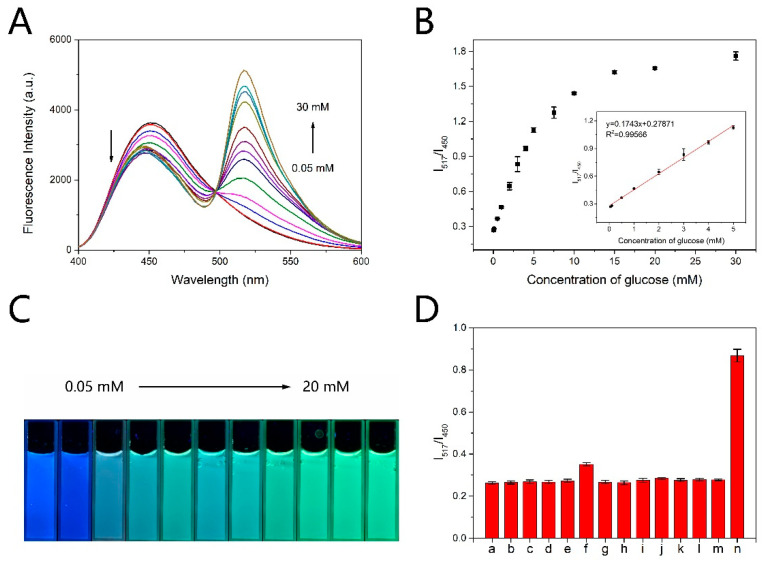
(**A**) Fluorescence spectra of CDs-PF probe after 3 min’s reaction with different concentration of glucose oxidation products. Excitation was at 365 nm. (**B**) Scatter diagram of I_517_/I_450_ versus the concentration of glucose. Inset: linear relation between I_517_/I_450_ and glucose concentration. (**C**) Fluorescence image of CDs-PF probe corresponding to (**A**). (**D**) Fluorescence intensity ratio (I_517_/I_450_) of CDs-PF probe after incubation with different interferents. (a)–(n) represent blank samples, Cl^−1^, K^+^, Na^+^, L-cysteine, D-galactose, glycine, glutathione, D-Fructose, L-ascorbic acid, urea, α-lactose, sucrose, D-glucose, respectively. Error bars in (**B**) and (**D**) are based on standard deviations (n = 3).

The short time detection method was applied to the ratiometric fluorescence detection of glucose. All the fluorescence intensities shown in the figures were obtained by the short time detection method. As shown in Figure 4A, with the concentration of glucose increasing, the fluorescence intensity at 517 nm gradually increased, while the fluorescence intensity at 450 nm gradually decreased. Moreover, with the increasing concentration of glucose the photographs of the mixed solution in cuvette exhibited a fluorescence color change from blue to green under UV light (365 nm) illumination (Figure 4C), which could be further applied to visual detection of glucose. The fluorescence intensity ratio (I_517_/I_450_) changes linearly with the concentration of glucose increasing from 0.05 mM to 5 mM (Figure 4B). The regression equation is y = 0.1743x + 0.27871 (R^2^ = 0.99566). The limit of detection (LOD) is estimated to be 17.19 μM according to 3σ rule. We can make the probe compatible with the physiological concentration range by diluting the plasma in practical use. Rapid detection and broad detection range make the probe comparable or superior to those reported methods (Table 1).

The selectivity of the probe for glucose was investigated. The common interferents with the same concentration as glucose including Cl^−^, K^+^, Na^+^, L-cysteine, D-galactose, glycine, glutathione, D-fructose, L-ascorbic acid, urea, α-lactose, sucrose, were added to the probe solution to detect the sensitivity. The concentration of glucose was 5 mM. Figure 4D showed that the ratio of the fluorescence intensity (I_517_/I_450_) was almost unchanged in the presence of interferents, and obviously increased upon addition of glucose. The results indicated the good selectivity of the probe.

### 3.7. Real Sample Detection

We also investigated the applicability of the probe for glucose detection in human blood samples. The recovery test of spiked samples was performed to evaluate the accuracy of the method. As shown in Table S2, the recovery was in the range of 96.67–104.33% with relative standard deviation below 2%. The results indicate that the probe and the proposed detection method are applicable for glucose detection in real samples.

## 4. Conclusions

In summary, we developed a ratiometric fluorescence probe based on the conjugation of CDs and PF for rapid and selective detection of glucose. The FRET-based ratiometric fluorescence detection can avoid instrument errors to some extent because of the self-calibration. By studying the kinetics of the reaction between PF and H_2_O_2_, a short time detection method was proposed and applied to CDs-PF probe for glucose detection. The method has potential for extending to other time-consuming detection in biosensing. The probe well realized rapid and linear detection of glucose in the range of 0.05 mM–5 mM with detection limits of 17.19 μM. It took only 3 min to complete a detection, even if the reaction is almost at the beginning stage. A lot of detections based on chemical reactions are slow, which makes the detections inefficient. Thus, the method we proposed is meaningful for rapid detection in slow reaction. Furthermore, the probe is insensitive to the common interferents and can be successfully applied to the detection of glucose in human serum with high accuracy. The numerous advantages indicate that the probe and the detection method have practical significance in glucose detection.

## Figures and Tables

**Table 1:** Comparison of glucose detection methods.

Probe	Linear Range (μM)	LOD (μM)	Ref
CuAA/Mg–N-CQDs	2–400	1.56	[46]
C-dots/Fe^2+^/VB1	0.5–450	0.074	[16]
AgNPs@PCN-224	5–80	0.078	[47]
ZnO TPs/MXene	50–700	17	[48]
Hierarchical sheet-like Ni-BDC/GCE	10–800	6.68	[9]
CDs-PF	50–5000	17.19	This work

## Data Availability

The data presented in this study are available on request from the corresponding author. The data are not publicly available due to privacy restrictions.

## Supplementary Materials

The following supporting information can be downloaded at: https://www.mdpi.com/article/10.3390/bios13020222/s1, Figure S1: FT-IR spectrum of PF, CDs, CDs-PF and CDs-Fluorescein. Top to down: PF, CDs, CDs-PF, CDs-Fluorescein; Figure S2: (A) XPS spectrum of CDs-PF. (B) The high resolution B1s spectrum; Figure S3: The emissions of CDs under excitation wavelength ranging from 320 nm to 420 nm; Figure S4: Fluorescence intensity of CDs under continuous UV light (365 nm) illumination. Fluorescence intensities were recorded every 5 min. Error bars are based on standard deviations (n = 3); Figure S5: Ratiometric fluorescence response of 0.0075mg/mL probe to 1mM H_2_O_2_. The dual emission fluorescence spectrum was recorded every 10 minutes after H_2_O_2_ was added; Figure S6: The spectra of (A) CDs and (C) fluorescein under different pH. The peaks at 490 nm are the scattering peaks of 490 nm excitation in (C). The emission peak of (B) CDs and (D) fluorescein under different pH. Error bars in (B) and (D) are based on standard deviations (n = 3); Figure S7: Ratiometric fluorescence response of the ratiometric probe to 5mM glucose. The blue and green lines were the spectra of the ratiometric probe before and after addition of glucose incubation soulution respectively. The reaction time was 3 minutes. Inset: corresponding fluorescence images under UV light (365 nm); Figure S8: Effect of different concentration of PF on fluorescence intensity in detection platform. Error bars are based on standard deviations (n = 3); Figure S9: Fluorescence decay curves of CDs-PF (black) and CDs-Fluorescein (blue); Figure S10: Linear relation between concentration and fluorescence intensity of fluorescein (generated from PF) in the low concentration range; Table S1. Fluorescence decay lifetimes τ and the relative fluorescence intensity percentages C for CDs-PF and CDs-Fluorescein. χ^2^ is the reduced Chi-Square value for each τ_avg_; Table S2. Results of glucose determination in real samples.
