# Supplementary material for: CDs-Peroxyfluor Conjugation for Ratiometric Fluorescence Detection of Glucose and Shortening Its Detection Time from Reaction Dynamic Perspective
Source: Biosensors (Basel). 2023 Feb 3;13(2):222. doi: 10.3390/bios13020222 (PMC9953814; doi:10.3390/bios13020222)
Supplement: Supplementary file 1 [file biosensors-13-00222-s001.zip › biosensors-2187981-supplementary.pdf]

biosensors

Supplementary Material

CDs-Peroxyfluor Conjugation for Ratiometric Fluorescence Detection of Glucose and Shortening Its Detection Time from Reaction Dynamic Perspective

Yangjie Li, Site Luo, Xin Wang, Yang He and Haihu Yu *

National Engineering Laboratory for Fiber Optic Sensing Technology, Wuhan University of Technology, Wuhan 430070, China
* Correspondence: hhyu@whut.edu.cn

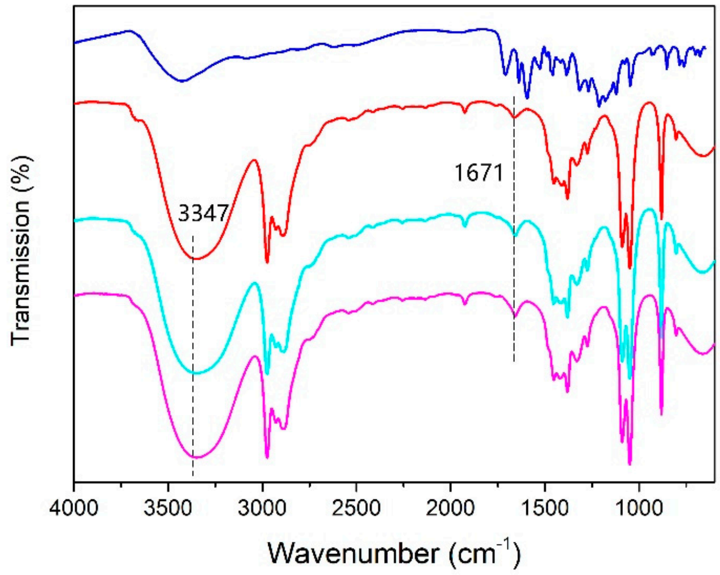

Figure S1. FT-IR spectrum of PF, CDs, CDs-PF and CDs-Fluorescein. Top to down: PF, CDs, CDs-PF, CDs-Fluorescein.

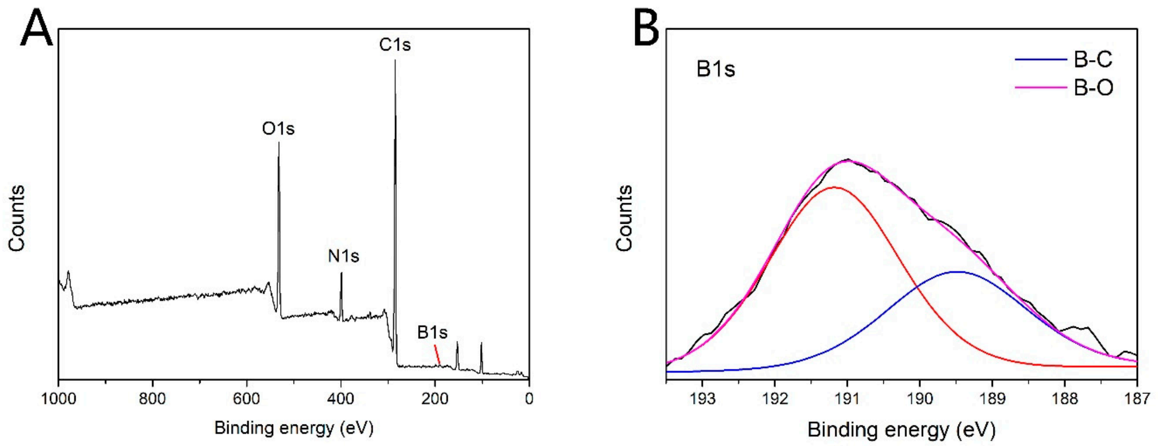

Figure S2. (**A**) XPS spectrum of CDs-PF. (**B**) The high resolution B1s spectrum.

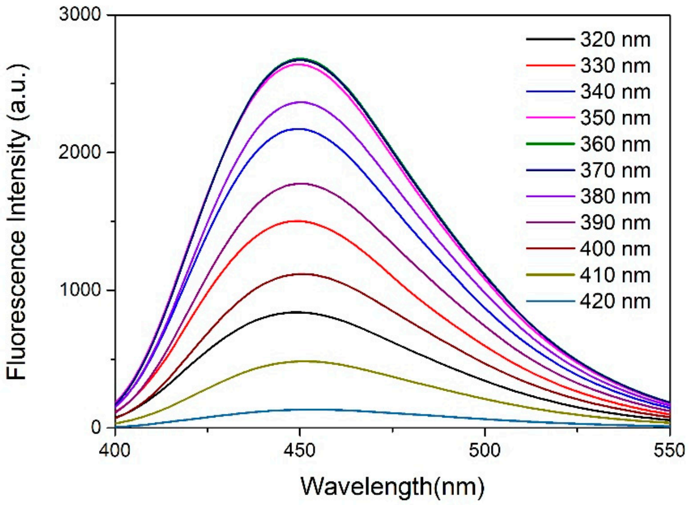

Figure S3. The emissions of CDs under excitation wavelength ranging from 320 nm to 420 nm.

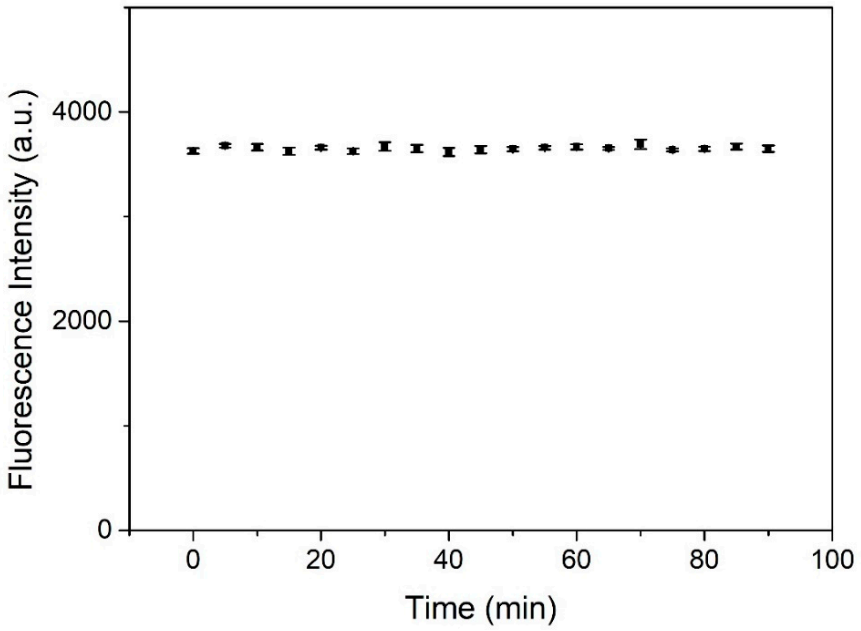

Figure S4. Fluorescence intensity of CDs under continuous UV light (365 nm) illumination. Fluorescence intensities were recorded every 5 min. Error bars are based on standard deviations (n = 3).

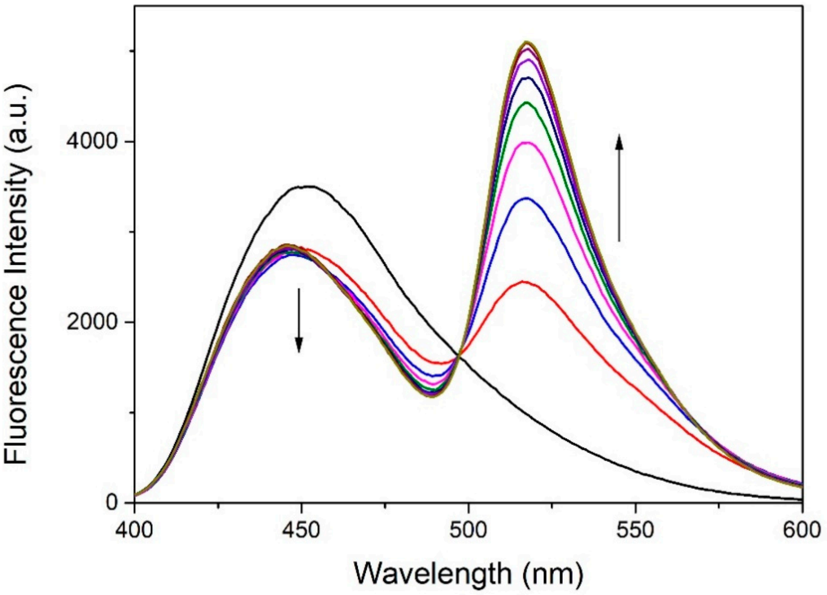

Figure S5. Ratiometric fluorescence response of 0.0075mg/mL probe to 1mM H_2O_2. The dual emission fluorescence spectrum was recorded every 10 minutes after H_2O_2 was added.

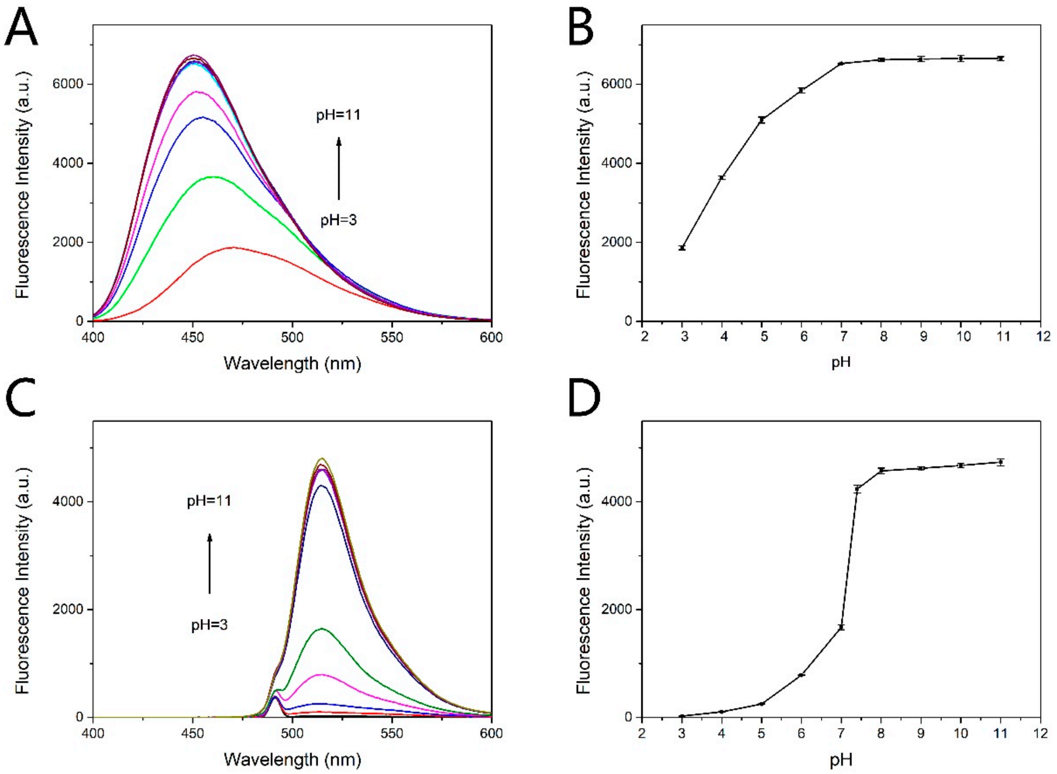

Figure S6. The spectra of (**A**) CDs and (**C**) fluorescein under different pH. The peaks at 490 nm are the scattering peaks of 490 nm excitation in (**C**). The emission peak of (**B**) CDs and (**D**) fluorescein under different pH. Error bars in (**B**) and (**D**) are based on standard deviations (n = 3).

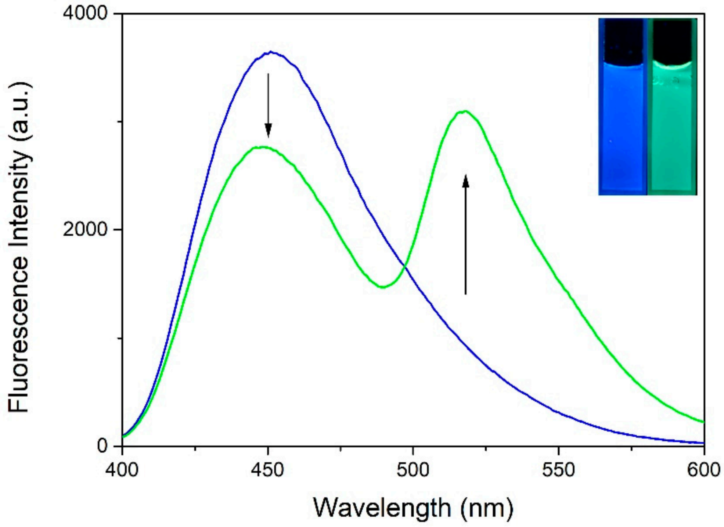

Figure S7. Ratiometric fluorescence response of the ratiometric probe to 5mM glucose. The blue and green lines were the spectra of the ratiometric probe before and after addition of glucose incubation soulution respectively. The reaction time was 3 minutes. Inset: corresponding fluorescence images under UV light (365 nm).

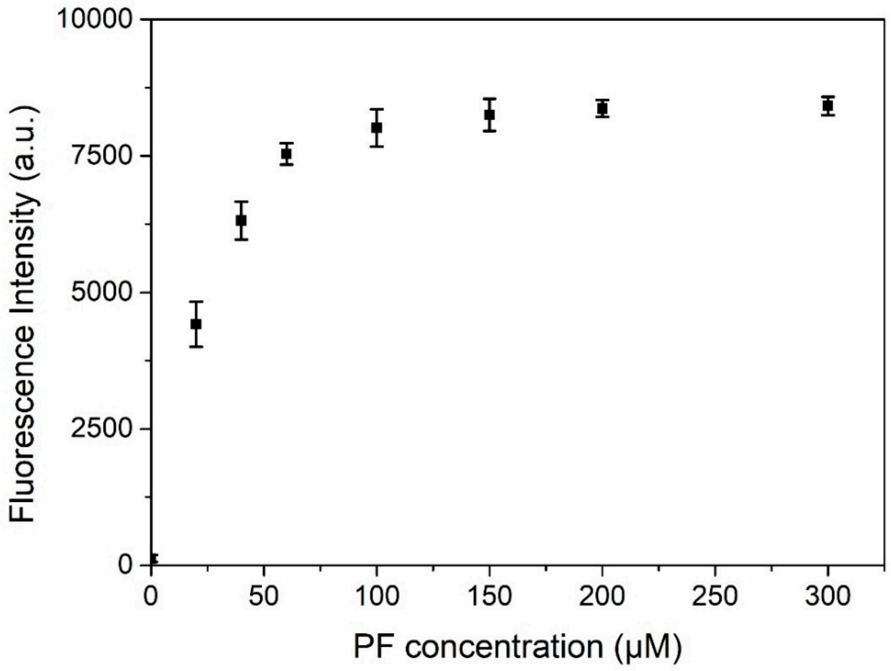

Figure S8. Effect of different concentration of PF on fluorescence intensity in detection platform. Error bars are based on standard deviations (n = 3).

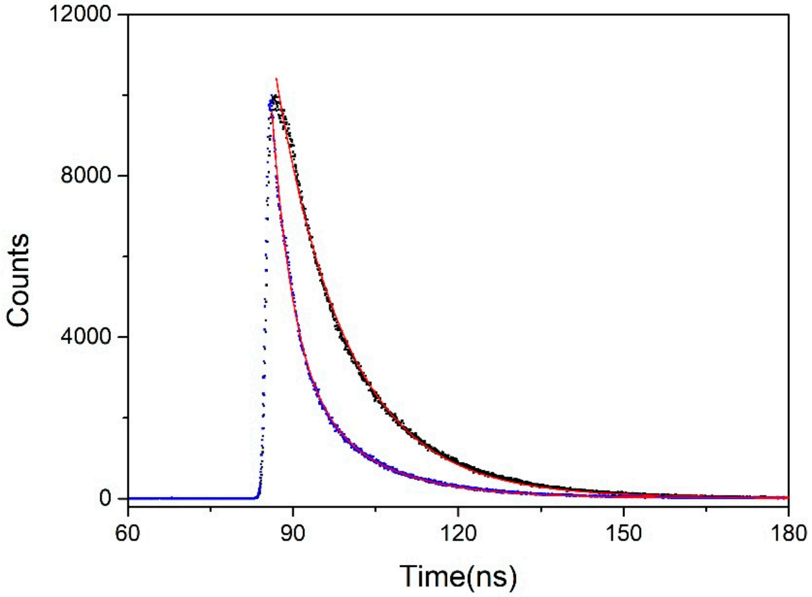

Figure S9. Fluorescence decay curves of CDs-PF (black) and CDs-Fluorescein (blue).

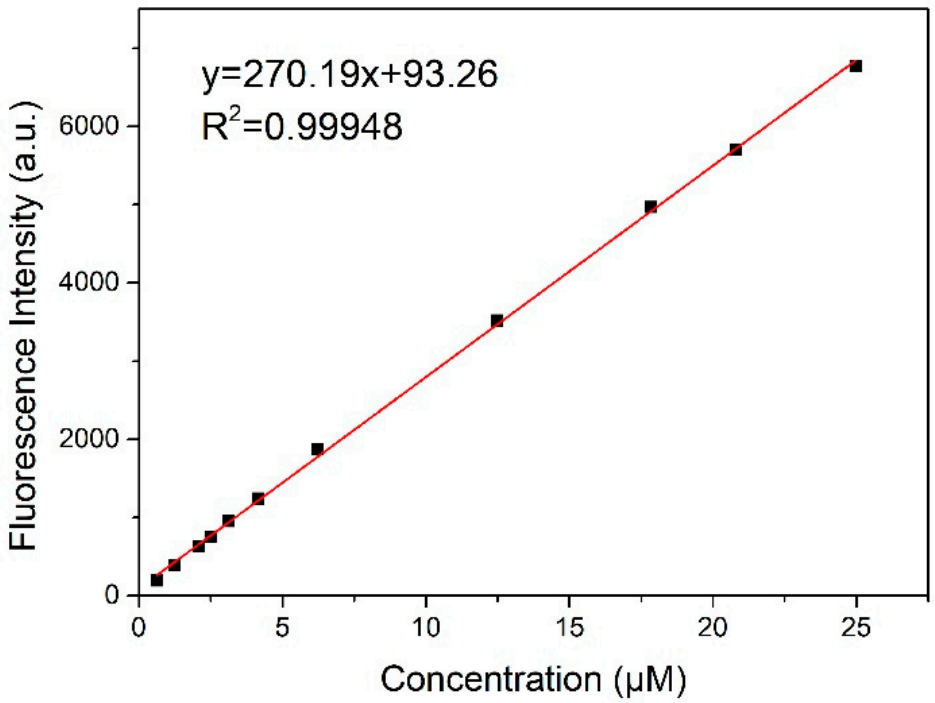

Figure S10. Linear relation between concentration and fluorescence intensity of fluorescein (generated from PF) in the low concentration range.

Table S1. Fluorescence decay lifetimes τ and the relative fluorescence intensity percentages C for CDs-PF and CDs-Fluorescein. χ^2 is the reduced Chi-Square value for each τ_{avg}.

Sample	τ_1 [ns]	C_1 [%]	τ_2 [ns]	C_2 [%]	τ_3 [ns]	C_3 [%]	τ_{avg}[ns]	χ^2
CDs-PF	10.27	36.34	16.53	31.35	1.82	32.31	13.16	1.16
CDs-Fluorescein	5.096	21.64	0.939	67.33	15.174	11.03	9.27	1.2

Table S2. Results of glucose determination in real samples.

Sample	Added (mM)	Found (mM)	Recovery (%)	RSD (%, n=3)
1	1	1.06	104.33	1.46
2	2	2.03	102.33	1.85
3	3	2.93	96.67	1.24
